# Progress in Laryngology

**Published:** 1900-09-22

**Authors:** 


### Progress in Laryngology.

*(Continued from page 405.)*

Roughton[4] considers that in most cases the treatment of antral empyema should be commenced by alveolar drainage, using a good-sized tube; if this method fails after a fair trial, the radical operation through the canine fossa should be performed. The external operation in frontal empyema should be urged when there is pain and other evidence of imperfect drainage, after a trial by removal of the anterior part of the middle turbinate and irrigation through the infundibulum. Tilley, in testing for pus in the antrum by transillumination, lays much stress upon the patient's subjective sensation of light in the eyes. He also supported alveolar drainage as a preliminary measure in all cases of antral suppuration; after this, in case of failure, the operation through the canine fossa should be advised. Want of success in this method is often due to imperfect curettage of the upper and inner region of the antrum, which is cellular in structure, and often closely associated with diseased anterior-ethnoidal cells.

De Roaldes[5] advocates strongly in chronic empyema of the antrum of Highmore, where alveolar drainage has failed, the operation known as the Caldwell-Luc method. In this procedure, the patient having been anæsthetised, the anterior third of the inferior turbinated bone of the affected side is removed with cutting forceps; the nasal fossa is then firmly packed with gauze. The upper lip is next inverted and retracted and an incision made horizontally, extending from just below the gingivo-labial fold near the frænum anteriorily, back to the root of the first molar. The periosteum is included and the flap dissected from the bone leaving the anterior wall bare. By means of a chisel and forceps an opening is then made through the canine fossa into the antral cavity large enough to admit the finger readily. After thoroughly curetting the antral cavity the gauze is removed from the nasal cavity and a finger placed on the site of the removed turbinate. A hole is now made by means of a chisel from the antral side into the nasal fossa sufficiently large to admit a finger. The flap is then replaced so as to cut off all communication with the mouth. De Roaldes states that he has cured all his patients requiring and submitting to this operation in from four to six weeks. He is convinced that "a more general application of the Caldwell-Luc method will prove it to be a most valuable operation in the treatment and cure of cases which too frequently have been the opprobrium of rhinology." Ward prefers to make the opening through the canine eminence instead of the fossa. Shirley thinks that the patient should never be left with only one opening and that in the nose; the alveolar opening should be left as well.

Ackland[6] has devised some instruments for the treatment of antral empyema by perforation through the alveolar ridge. The instruments comprise: (1) A borer; (2) a measurer to ascertain the depth of bone traversed; (3) the tube carrier; (4) the tube; (5) a two-way nozzle which exactly fits the tube, and which is used for the purposes of irrigation.

Harrison Griffin[7] considers that if the inferior turbinated bone is found to lie close to the septum after the mucous membrane has been treated with cocaine and supra-renal capsule extract there must be an enlargement of the bone itself, and he does not hesitate to operate upon every case in which after the application of these drugs the septo-turbinated space is partly or entirely obliterated. To guard against secondary hæmorrhage he prescribes 10 grs. of quinine sulph. and 20 grs. of bromide each night at bedtime for a week or two previous to the operation, Warburg's tincture also being given three times daily before each meal. After the part has been treated with supra-renal capsule extract the turbinated body is removed by means of a small saw introduced well back under the bone. The cut is then made from below upwards and inwards towards the septum. The bone is removed with forceps. Griffin, who has performed this operation over a thousand times, asserts that in each case the patient was greatly relieved. He is so impressed with it that he recommends it also in the case of recurring polypi of the nose.

Sir Felix Semon,[8] in lecturing upon Nasal Reflex Neuroses, concludes that it would be folly or something worse to condemn the whole doctrine of nasal reflex neuroses as fallacious and misleading, but he is convinced that "the frequency and importance of the influence of the nasal mucous membrane upon nervous phenomena at distant parts of the body has been grossly exaggerated," and that the mechanism of these processes is not understood. Juracz says that in discussing the pathogenesis of nasal reflex neuroses we enter upon a region of neurology in which a reply to the various questions cannot be given. The reflex apparatus in the nose is of a very complex nature. The centripetal and centrifrugal paths are very numerous, whilst the nature and localisation of the irritation are not always well known. Little that is definite can be said on our existing knowledge, and we are constrained to make a number of hypotheses which may at least approximately give us an idea of the origin of nasal reflex neuroses.

Dr. Burell[9] asserts that the conditions of the nose most liable to produce reflex phenomena in other organs are those in which the septum and turbinate bodies are in contact. He cites many cases cured by nasal operations.

Hartman,[10] from his investigations on the anatomy of the frontal sinus and anterior-ethmoidal cells, shows from their development that it is not sufficient to remove the floor of the frontal sinus, but the floor of the frontal, *i.e.*, the anterior-ethmoidal, cells should be removed as well, in order to obtain a free communication between the frontal sinus and the nose. He advises that an opening be made into the frontal sinus, and another into the frontal cells, through the inner wall of the orbit, and that the walls of the frontal cells be removed with forceps.

Beaman Douglas,[11] investigating the effect of the galvano-cautery knife, finds that it forms a cone of burned tissue, the effect being greatest at the periphery of the cone, the sub-epithelial limiting membrane being extensively affected; in some cases tissue away from the cauterised part was affected by the heat. Specimens taken 100 hours after cauterisation showed a greater amount of blood in the vessels near the burn than in those at a distance from the cauterised area, and there were also "a few thrombosed vessels"; this was important in showing a serious result in a tissue removed from the cautery point. Repair had commenced, and there was no inflammation. In specimens removed 360 hours after cauterisation, the new tissue was already filling up the gap. Dr. Douglas concludes that the cautery should never be used superficially over a large area, but should be rapidly introduced into the deeper structures, and never drawn forwards or backwards. When contact is made the cautery point should scarcely be heated. He further concludes that the upper nasal region, the nasal roof, the ethmoid region, the outer nasal wall, the middle turbinated body, except its anterior and posterior ends, should never be approached with the cautery, because of the difficulty in controlling its effects. The peculiar œdema following its use renders it particularly dangerous about the uvula, faucial pillars, the arytenoid region, and aryteno-epiglottal folds. He considers it applicable to soft structures, especially those composed of round cells. It is unsuitable to prevent the return of polypi, whilst in malignant growths it should never be used. Harris finds the galvano-cautery extremely useful, and, in spite of its abuse, there was no good reason for specialists discarding it. Quinlan, Curtis, Frendenthal, and Myles all advocate extreme caution in the use of the cautery upon the middle turbinate body.

[4] Lancet, April 14, 1900. [5] New York Med. Jour., Jan. 6, 1900. [6] Brit. Med. Jour., June 2, 1900. [7] New York Med. Jour., Feb. 24, 1900. [8] Clinical Jour., Feb. 7, 1900. [9] Post Graduate, Feb., 1900. [10] Jour. of Laryngology, Feb., 1900. [11] Laryngoscope, 1900.

